# A Cross‐Sectional Cohort Study of the Effects of FGF23 Deficiency and Hyperphosphatemia on Dental Structures in Hyperphosphatemic Familial Tumoral Calcinosis

**DOI:** 10.1002/jbm4.10470

**Published:** 2021-03-22

**Authors:** Alisa E Lee, Emily Y Chu, Pamela J Gardner, Olivier Duverger, Amanda Saikali, Sean K Wang, Rachel I Gafni, Iris R Hartley, Kelly G Ten Hagen, Martha J Somerman, Michael T Collins

**Affiliations:** ^1^ National Institute of Dental and Craniofacial Research, National Institutes of Health Bethesda MD USA; ^2^ National Institute of Arthritis and Musculoskeletal and Skin Diseases, National Institutes of Health Bethesda MD USA; ^3^ Eunice Kennedy Shriver National Institute of Child Health and Human Development, National Institutes of Health Bethesda MD USA

**Keywords:** BONE MICRO‐COMPUTED TOMOGRAPHY (μCT), BONE QUANTITATIVE COMPUTED TOMOGRAPHY (QCT), DENTAL BIOLOGY, DISORDERS OF CALCIUM/PHOSPHATE METABOLISM, FIBROBLAST GROWTH FACTOR 23 (FGF23), MOLECULAR PATHWAYS—DEVELOPMENT, PARATHYROID HORMONE, VITAMIN D

## Abstract

Hyperphosphatemic familial tumoral calcinosis (HFTC) is a rare autosomal recessive disorder caused by mutations in *FGF23*, *GALNT3*, *KLOTHO*, or FGF23 autoantibodies. Prominent features include high blood phosphate and calcific masses, usually adjacent to large joints. Dental defects have been reported, but not systematically described. Seventeen patients with HFTC followed at the National Institutes of Health underwent detailed clinical, biochemical, molecular, and dental analyses. Studies of teeth included intraoral photos and radiographs, high‐resolution μCT, histology, and scanning electron microscopy (SEM). A scoring system was developed to assess the severity of tooth phenotype. Pulp calcification was found in 13 of 14 evaluable patients. Short roots and midroot bulges with apical thinning were present in 12 of 13 patients. Premolars were most severely affected. μCT analyses of five HFTC teeth revealed that pulp density increased sevenfold, whereas the pulp volume decreased sevenfold in permanent HFTC teeth compared with age‐ and tooth‐matched control teeth. Histology revealed loss of the polarized odontoblast cell layer and an obliterated pulp cavity that was filled with calcified material. The SEM showed altered pulp and cementum structures, without differences in enamel or dentin structures, when compared with control teeth. This study defines the spectrum and confirms the high penetrance of dental features in HFTC. The phenotypes appear to be independent of genetic/molecular etiology, suggesting hyperphosphatemia or FGF23 deficiency may be the pathomechanistic driver, with prominent effects on root and pulp structures, consistent with a role of phosphate and/or FGF23 in tooth development. Given the early appearance and high penetrance, cognizance of HFTC‐related features may allow for earlier diagnosis and treatment. © 2021 The Authors. *JBMR Plus* published by Wiley Periodicals LLC. on behalf of American Society for Bone and Mineral Research.

## Introduction

Hyperphosphatemic familial tumoral calcinosis (HFTC; Online Mendelian Inheritance in Man [OMIM]: 211900) is a rare autosomal recessive disorder caused by deficiency of active fibroblast growth factor 23 (FGF23), a hormonal regulator of phosphate and vitamin D homeostasis.^(^ ^1^ ^)^ FGF23 is produced by osteoblasts and osteocytes in response to high blood phosphate and 1,25‐dihydroxy vitamin D [1,25(OH)_2_D].^(^ ^2^, ^3^ ^)^ FGF23 binds to FGF receptor‐1 (FGFR1) and coreceptor KLOTHO to downregulate sodium/phosphate cotransporters NPT2a and NPT2c and 25‐hydroxyvitamin D‐1α‐hydroxylase, which converts inactive 25‐hydroxyvitamin D to 1,25(OH)_2_D, thereby inhibiting phosphate reabsorption and active vitamin D formation in renal proximal tubules.^(^ ^4^, ^5^, ^6^ ^)^

Intact FGF23 (iFGF23) undergoes posttranslational O‐glycosylation by the enzyme UDP‐GalNAc:polypeptide N‐acetylgalactosaminyltransferase 3 (GALNT3), which is thought to stabilize and protect iFGF23 from furin degradation.^(^ ^7^ ^)^ Without glycosylation, iFGF23 is cleaved into physiologically inactive C‐ and N‐terminal fragments. Therefore, HFTC is a disorder of iFGF23 deficiency.^(^ ^8^ ^)^ Causes include mutations in *FGF23*, *GALNT3*, or *KLOTHO*.^(^ ^9^, ^10^, ^11^ ^)^ FGF23 autoantibodies cause an acquired form of tumoral calcinosis.^(^ ^12^ ^)^

HFTC manifests across a broad clinical spectrum, including painful subcutaneous masses, calcification around joints, hyperostosis of long bone diaphyses, systemic inflammation, ocular involvement, and dental pathology.^(^ ^13^ ^)^ Most diagnosed patients develop symptoms by 2–13 years of age.^(^ ^14^ ^)^ Because of its rarity, phenotypic variability, and unfamiliarity with signs and symptoms, HFTC is likely underdiagnosed; patients may also carry mutations that do not have detectable clinical symptoms.

Dental pathology may be the most common phenotypic feature of HFTC.^(^ ^1^ ^)^ Several case reports and small series have described short, bulbous roots, obliteration of pulp chambers and canals, and periapical radiolucencies.^(^ ^15^, ^16^, ^17^, ^18^, ^19^ ^)^ Hyperphosphatemia is speculated to be the primary contributor,^(^ ^20^ ^)^ but molecular and cellular mechanisms have not been explored. To better define the spectrum, natural history, and pathophysiology of HFTC‐associated dental findings, we performed a detailed analysis of a cohort of 17 patients.

## Patients and Methods

### Patients

The study was approved by the National Institute of Dental and Craniofacial Research Institutional Review Board. All patients or legal guardians provided informed consent. Patients underwent clinical, biochemical, genetic, and dental examinations. This study was performed in accordance with STROBE (Strengthening the Reporting of Observational Studies in Epidemiology) guidelines for human observational investigations.

### Dental phenotype scoring

**Fig 1:**
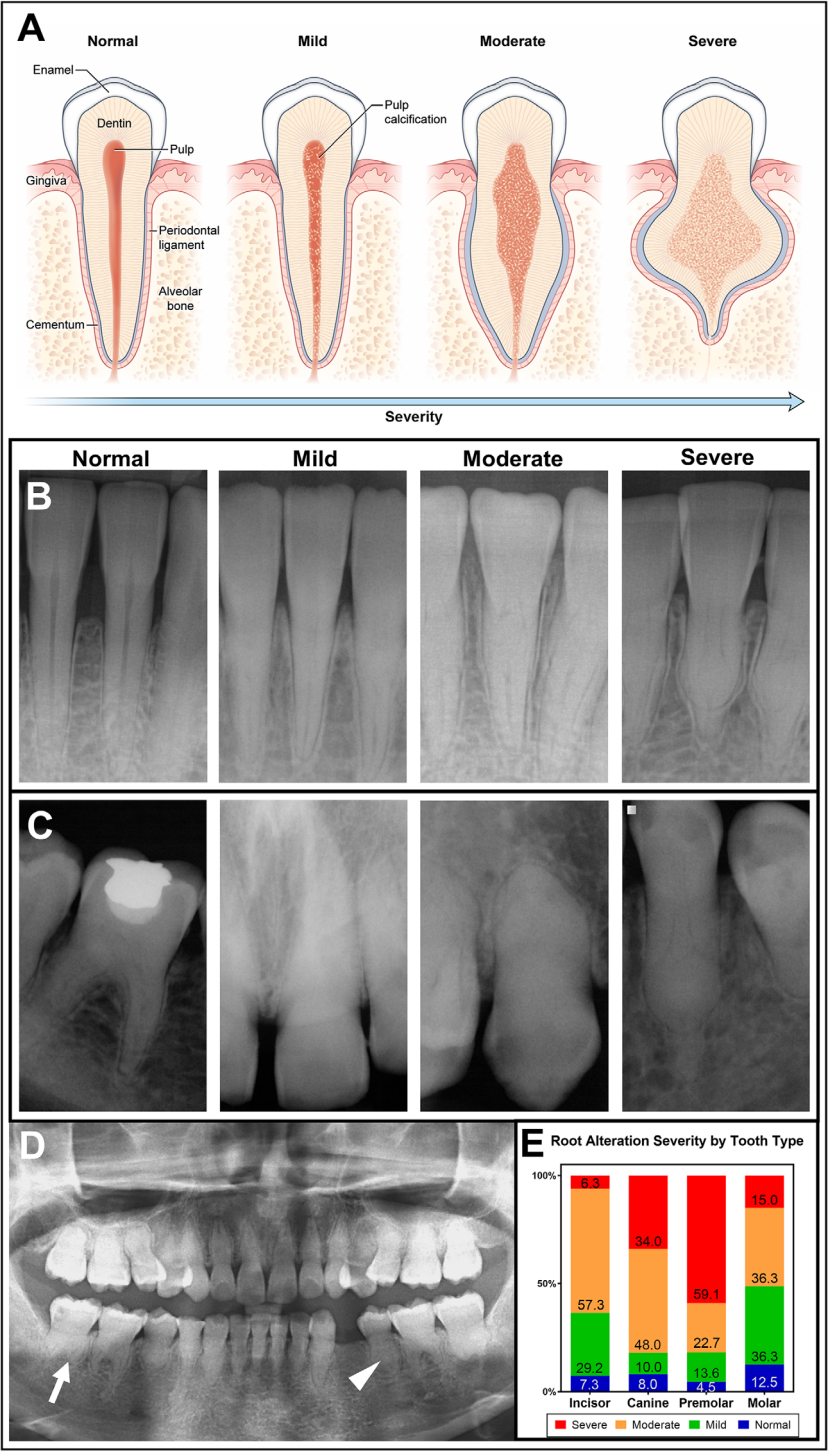
Spectrum of dental phenotypes in hyperphosphatemic familial tumoral calcinosis (HFTC). (*A*) Schematic drawing of categorization used to score the degree of dental severity in patients with HFTC. Normal tooth has healthy pulp without any calcifications. Mild phenotype shows partial calcification of pulp with unaltered root shape. Moderate phenotype shows root bulging with pulp calcification. Severe phenotype shows shortened thistle‐shaped root with extensive pulp calcification. (*B*) Periapical radiographs of mandibular incisors in order of normal (healthy 47‐year‐old male), mild (patient 5), moderate (patient 16), and severe (patient 6) phenotypes. (*C*) Periapical radiographs show a wide spectrum of dental phenotypes in a single patient (patient 4). From left to right: tooth #30, 9, 6, and 21. (*D*) Panoramic radiograph of the patient 6 with HFTC at age 20 years. Short bulbous root (arrow) with partial to complete pulp obliteration (arrowhead) is observed in all teeth. (*E*) The percentages of all the teeth affected in all the patients are shown. Premolars are most severely affected, followed by canines, molars, and incisors. The severity of root alteration between tooth types was significantly different at *p* < 0.0001 using the χ^2^ test. A more detailed graph by tooth type is provided in Supplementary Information Figure S1.

Dental panoramic and/or periapical radiographs and intraoral photographs were taken for 14 and 11 patients, respectively. For patient 1, radiographs taken prior to enrollment were retrieved. Dental radiographs were not available for patients 9 and 14 because of young age and/or lack of cooperation. Exfoliated/extracted primary and permanent teeth were collected from five patients and four age‐ and tooth‐matched healthy controls. Dental radiographs were independently read by a dentist (PJG) and a trained dental student (AEL) to determine the severity of root morphology using a predefined scoring system (Fig. 1). The reliability of the scoring system was examined by intra‐ and interrater reliability analyses. The intrarater reliability ranged from 0.75 to 1, and the interrater reliability between two dentists ranged from 0.52 to 1. Third molars were excluded.

### Calcification scoring

Soft tissue calcification was scored on a semiquantitative system developed by the investigators. Patients were assigned to groups ranging from 0 (no calcification) to 3+ (marked/diffuse calcification involving all three anatomical compartments: craniofacial/neck, abdomen/pelvis, and axial skeleton), and 1+ and 2+ (involving one or two anatomical compartments, respectively). Vascular calcification was scored as yes/no based on its presence on radiographs.

### Biochemical

Biochemical evaluation (phosphate, calcium, vitamin D, and PTH) were performed by standard techniques. Intact and C‐terminal FGF23 (cFGF23) were measured in multiple ELISAs (Kainos Laboratories, Immutopics Quidel, or the Mayo Clinic in Rochester, MN, USA).

### Genetic analysis

*GALNT3* sequencing was performed for patients 11, 16, and 17 in a commercial laboratory (Connective Tissue Gene Tests [CTGT]) and for patients 1–6, 8, 13, and 14 in a research laboratory using Multiplex PCR kit (QIAGEN) and ABI PRISM 3100 Genetic Analyzer (Applied Biosystems). *FGF23* sequencing was performed in a commercial laboratory (GeneDx). Patient 9 was found to have a triplication within chromosome 13, which likely altered the expression of *KLOTHO* (Greenwood Genetic Center). The National Center for Biotechnology Information reference sequences and OMIM numbers are in Supplementary Information Table S1.

### Micro‐computed tomography

Teeth samples were analyzed using a μCT 50 scanner (Scanco Medical) at 70 kVp, 76 μA, 0.5 Al filter, 900‐ms integration, and 10‐μm voxel size. AnalyzePro 1.0 (AnalyzeDirect) was used to reconstruct and analyze images. Images were anatomically oriented and calibrated to a linear standard curve created using hydroxyapatite phantoms with known densities. Enamel was segmented ≥1650 mg HA/cm^3^, and dentin/cementum at 600–1649 mg HA/cm^3^. HFTC dentin and cementum were indistinguishable by density, so dentin and cementum were combined for analysis. Pulp was segmented using a combination of manual and semiautomatic functions. Heat maps were generated for density visualization.

### Histology

Teeth were fixed in formalin for 48 h and hemisected axially using an Isomet slow‐speed diamond saw (Buehler). Half were demineralized in acetic acid/formalin/sodium chloride, embedded in paraffin, sectioned buccolingually at 5‐μm thickness, and stained with hematoxylin and eosin and Masson's trichrome. Images were acquired using an Aperio CSO Slide Scanner (Leica Biosystems).

### Scanning electron microscopy

Hemisected teeth were embedded in acrylic, ground on 320‐ and 1200‐grit sandpaper, and polished using 1‐μm and one‐quarter–μm diamond pastes. Samples were etched with 37% phosphoric acid for 10 s, and fixed sequentially in 1% glutaraldehyde overnight and 1% osmium tetroxide for 2 h. Samples were dehydrated in ethanol, incubated in hexamethyldisilazane, and air‐dried. Samples were mounted on aluminum specimen mount stubs (Electron Microscopy Sciences), coated with 5‐nm gold, and analyzed using the Field Emission Scanning Electron Microscope S4800 (Hitashi) at 10 kV.

### Statistical analysis

Data were analyzed by two‐tailed unpaired *t* tests (GraphPad Prism 8.4 software; GraphPad). Data are expressed as mean ± SD. *p* < 0.05 was considered statistically significant. Incidence of root abnormalities by tooth type and severity were compared using a χ^2^ test (SAS 9.4 TS1M6; SAS Institute). *p* < 0.05 was considered statistically significant.

## Results

### Summary of National Institutes of Health cohort

A cohort of 17 patients with HFTC (6 males, 11 females) was seen at the National Institutes of Health (NIH) between 2007 and 2020 (Table 1). Eight had compound heterozygous *GALNT3* mutations; three were novel. Four had homozygous *GALNT3* mutations, one novel. Patient 12 had compound heterozygous *FGF23* mutations, one novel. Patient 10 had neutralizing FGF23 autoantibodies. Patient 9 was found to have a chromosome 13 triplication in the *KLOTHO*‐containing region. Patients 7 and 15 had unknown but presumably genetic causes. Predicted protein changes are provided in Supplementary Information Table S2.

**Table 1:** Demographic, Dental, Clinical, Biochemical, and Genetic Findings in Hyperphosphatemic Familial Tumoral Calcinosis (HFTC) Cohort at Initial Visit

Patient	Age at symptom onset/current age (y)	Length of follow‐up (y)	Gender	Dental calcification^a^	Vascular calcification	Soft tissue calcification^b^	Phosphate^c^ (age‐specific normal range mg/dl)	FGF23		Genetic mutation^e^		Treatment
								Intact (22–63 pg/ml)	C‐term^d^ (≤230 RU/ml)	Gene	DNA change	
1	12/48	12	F	Y	N	1+	↑ 7.3 (2.5–4.8)	↓ 17	↑ 1210	*GALNT3*	c.1312C>T, c.1774C>T	ACZ, NC
2^f^	5/17	11	F	Y	Y	2+	↑ 8.8 (3.1–5.5)	34	↑ 2880	*GALNT3*	c.516‐2A>T, c.260_266del	SEV, ACZ, PB, AL, CAN
3^f^	9/15	7	F	Y	N	0	↑ 6.8 (3.1–5.5)	39	↑ 1985	*GALNT3*	c.516‐2A>T, c.260_266del	SEV, ACZ, PB
4^g^	10/38	9	M	Y	Y	3+	↑ 5.5 (2.5–4.8)	↓ 8^j^	↑ 660	*GALNT3*	c.1584dup	SEV, ACZ, PB, NC, AL, TT, ANA, CAN
5^g^	None/25	6	M	Y	N	0	↑ 5.5 (2.5–4.5)	39	↑ 971	*GALNT3*	c.1584dup	n/a
6	3/20	8	F	Y	N	1+	↑ 6.6 (3.1–5.5)	38^j^	↑ 884	*GALNT3*	c.516‐2A>T, c.1524+5G>A	SEV, ACZ, PB
7	45/63	6	F	N	Y	3+	4.8 (2.5–4.8)	46^j^	↑ 1190	Unknown	n/a	SEV, ACZ, PB
8	1/12	5	F	n/a	N	2+	↑ 7.0 (3.0–5.7)	22	↑ 1031	*GALNT3*	c.746_749del, c.892del	SEV, ACZ, PB
9	6 mo/19	6	F	n/a^i^	N	2+	↑ 5.3 (2.5–5.1)	42	66	*KLOTHO*	arr 13q13.1q14.3(32,887,503‐53,157,340)x4 dn^k^	n/a
10	3 wk/12	5	M	Y	N	2+	↑ 7.2 (3.2–6.3)	↑ 13,000	↑ 33,000	Autoimmune	n/a	SEV, ACZ, PB, TT
11	10/47	4	M	Y	Y	3+	↑ 5.4 (2.5–4.5)	↓ 19	↑ 1420	*GALNT3*	c.766G>C^k^	SEV, ACZ, PB
12	11/46	3	M	Y	Y	2+	↑ 6.5 (2.5–4.5)	41	↑ 890	*FGF23*	c.211A>G, c.107G>A^k^	SEV, ACZ
13^g^	8/27	2	F	Y	N	2+	↑ 6.4 (2.5–4.5)	↓ 21	↑ 1470	*GALNT3*	c.1584dup	n/a
14^f^	None/2	1	M	n/a	N	0	↑ 7.4 (3.1–6.0)	23	↑ 2260	*GALNT3*	c.516‐2A>T, c.260_266del	SEV
15	7/53	1	F	Y	Y	3+	↑ 5.8 (2.5–4.5)	↓ 16	↑ 1910	Unknown	n/a	LC, ACZ, ANA, SEV
16	4/12	1	F	Y	N	2+	↑ 7.1 (3.0–5.7)	23	↑ 1860	*GALNT3*	c.985G>A^k^, c.1677_1680dup^k^	SEV, ACZ, AL, TT
17	10/74	1	F	Y	Y	2+	↑ 5.4 (2.5–4.5)	31	↑ 2450	*GALNT3*	c.746_749del, c.926T>G^k^	SEV
**Range** ^**h**^	1–45/2–74	1–12	**Prevalence** ^**h**^				4.8–8.8	8–46	660–2880			
**Mean** ^**h**^	10.4/33.3	5.1	5 M 10 F	12/13	7/15	12/15	6.4	27.8	1538.1			
**Median** ^**h**^	9/27	5					6.5	23	1420			

Abbreviations: ACZ, acetazolamide; AL, aluminum hydroxide; ANA, anakinra; CAN, canakinumab; FGF23, fibroblast growth factor 23; GALNT3, UDP‐GalNAc:polypeptide N‐acetylgalactosaminyltransferase 3; HFTC, hyperphosphatemic familial tumoral calcinosis; LC, lanthanum carbonate; n/a, not available; NC, nicotinamide; PB, probenecid; SEV, sevelamer; TT, topical thiosulfate.

^a^ More detailed dental data provided in Supplementary Information Table S3.

^b^ Soft tissue calcification grading: 0 normal – 3+ severe.

^c^ To convert phosphate to mmol/L, multiply by 0.323.

^d^ C‐terminal FGF23 [3M–17Y ≤230, ≥18 Y ≤ 180 RU/mL].

^e^ More genetic data included in Supplementary Information Table S2.

^f^ Patients 2, 3, and 14 are siblings.

^g^ Patients 4, 5, and 13 are siblings.

^h^ Calculated excluding patients 9 and 10.

^i^ Dental radiographs were not available. An exfoliated primary tooth had normal pulp morphology, but increased pulp density (200.8 mg HA/cm^3^) by μCT analysis.

^j^ Some iFGF23 were measured by Kainos Laboratories. The rest were measured by Immutopics Quidel.

^k^ Novel mutation.

**Fig 2:**
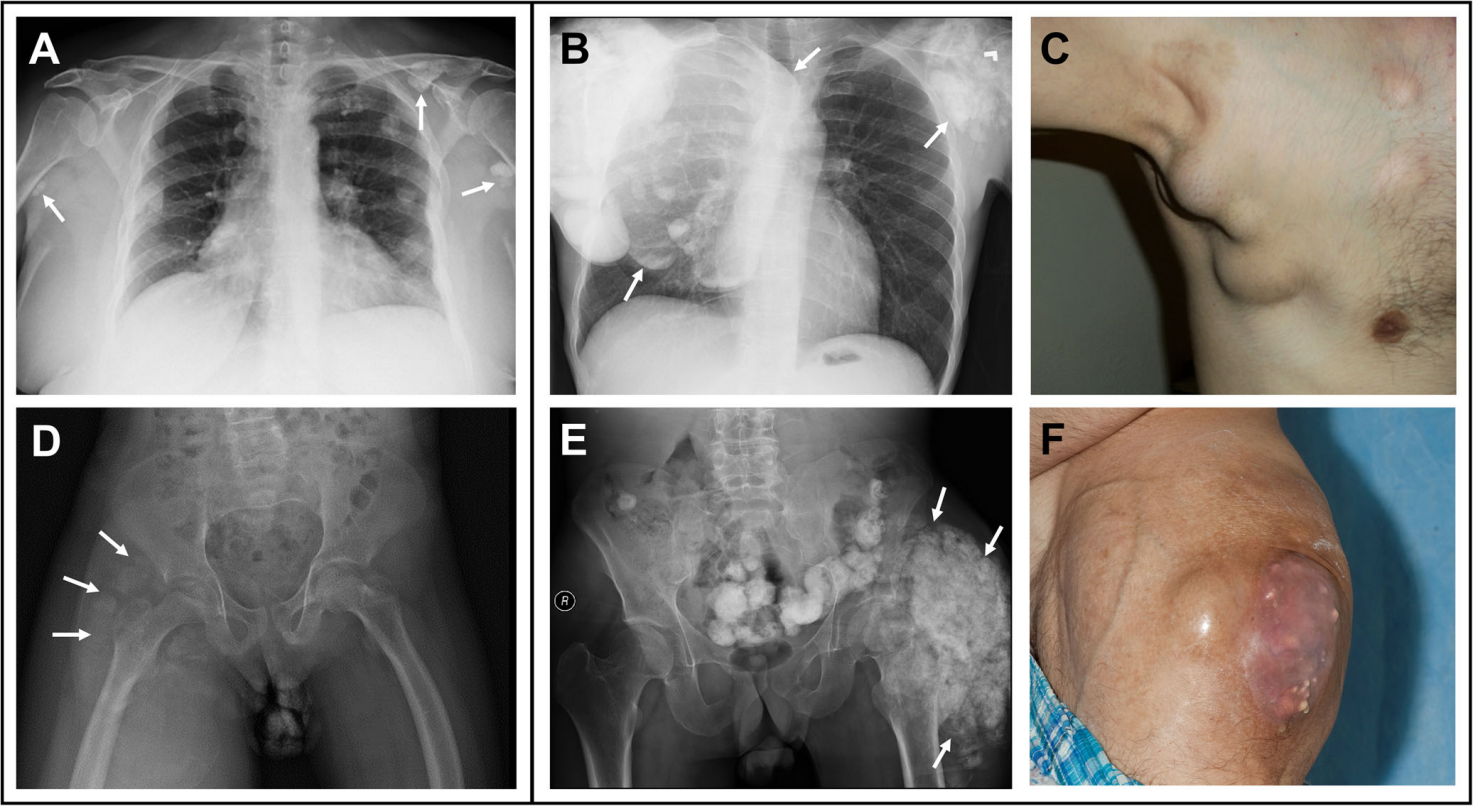
Examples of soft tissue calcifications in hyperphosphatemic familial tumoral calcinosis (HFTC). (*A*) Chest radiograph example of soft tissue calcification (arrows) score 1+ in Table 1. (*B*) Chest radiograph example of soft tissue calcification (arrows) score 3+. (*C*) Gross image of the axilla and chest calcinosis score 3+. (*D*) Pelvic radiograph example of soft tissue calcification (arrows) score 1+. (*E*) Pelvic radiograph shows extensive calcification of the left hip (arrows) receiving score 3+. (*F*) Gross image of hip calcinosis score 3+.

A wide genotype‐independent phenotypic spectrum exists, as found in the affected brothers (patients 4 and 5) with the same mutation and similar phosphate levels. Patient 4 was severely affected with tibial hyperostosis at age 10 years, which was misdiagnosed as osteomyelitis, and multiple, diffuse painful calcifications surrounding his shoulder, elbow, hip, and buttocks, and visceral and vascular calcifications starting at age 14 years (Fig. 2). Despite the lack of clinical symptoms or calcifications, his brother (patient 5) had an elevated C‐reactive protein, suggesting ongoing, subclinical calcification causing systemic inflammation. Elevated blood phosphate concentrations were present in most patients at 6.4 ± 1.0 mg/dl (normal: adults 2.5–4.5, children 3.0–5.7 mg/dl). Except for the patients with autoantibodies or *KLOTHO* mutation, all had low or normal iFGF23 and significantly elevated cFGF23.

### Classification and spectrum of dental phenotypes

Patients had short roots, pulp calcification, midroot bulges with apical thinning, and enamel defects. To quantify the severity of root alteration, a scoring scale was developed (Fig. 1*A*). Each tooth was scored from 1 to 4 (normal, mild, moderate, or severe, respectively; Supplementary Information Table S3; cohort average 2.9 ± 0.7). Periapical and panoramic radiographs were used to classify each tooth (Fig. 1*B*). Although the entire spectrum could be observed in a single patient (Fig. 1*C*), some had only moderate and severe phenotypes (Fig. 1*D*). Premolars were most severely affected, followed by canines, molars, and incisors (Fig. 1*E*; Supplementary Information Fig. S1). The severity of root alteration between tooth types were significantly different at *p* < 0.0001 using the χ^2^ test. Of patients with dental radiographs, 13 out of 14 (93%) showed partial to complete pulp obliteration. Notably, the patient without pulp obliteration (patient 7) had no identifiable *FGF23* or *GALNT3* mutation, and findings were inconsistent with a *KLOTHO* mutation. Of the 10 patients with *GALNT3* mutations and comprehensive dental exams, 100% had shortened, thistle‐shaped roots and pulp obliteration.

Other findings, not observed in all patients and probably unrelated to HFTC, were seen. White, chalky enamel was observed in patients 2 and 6, possibly caused by mild fluorosis (Supplementary Information Fig. S2). Gray enamel discoloration was observed in patients 1, 7, and 12. Patient 7 also had moderate enamel hypoplasia and facial surface pitting.

### Reduced pulp volume and increased density in HFTC

**Fig 3:**
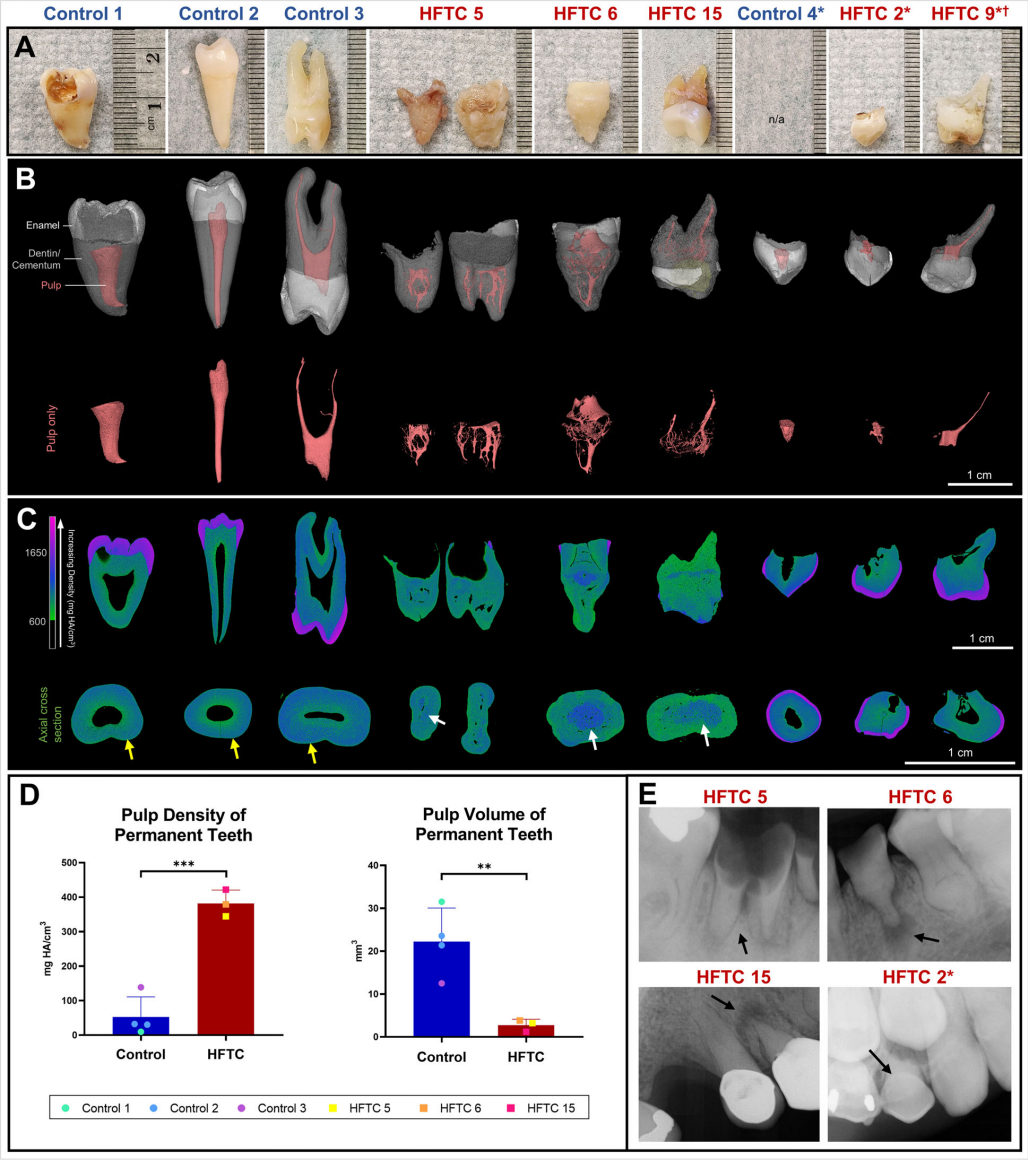
Clinical images and three‐dimensional (3D) reconstruction of the teeth of patients with hyperphosphatemic familial tumoral calcinosis (HFTC) reveal abnormal root and pulp. (*A*) Images of selected control and HFTC teeth samples. HFTC teeth have shorter roots and more irregular root surfaces. (*B*) 3D reconstructions with segmentation (see Patients and Method section) of control and HFTC teeth are shown (white = enamel, gray = dentin/cementum, pink = pulp, yellow = composite buildup). Pulp (pink) from 3D reconstructions shown in (*B*) are highlighted. Although control teeth have single pulp chambers, HFTC teeth show reduced pulp chamber and canals with capillary‐like morphology. (*C*) Longitudinal and cross‐sectional heat maps illustrate differences in density distribution. Color bar shows increasing density (green to purple). In HFTC teeth, dentin and cementum layers are indistinguishable. Areas of high density are found towards the periphery of root in control (yellow arrows) versus towards the core in HFTC teeth (white arrows). The majority of pulp has been replaced in HFTC teeth. Density of primary HFTC teeth does not appear to be as severely affected. (*D*) The mean pulp density was sevenfold higher in permanent HFTC teeth than in controls (382 ± 39 vs 53 ± 58 mg HA/cm^3^, *p* = 0.0004). The mean pulp volume was sevenfold lower in permanent HFTC teeth than in control (3 ± 1 vs 22 ± 8 mm^3^, *p* = 0.0088). (*E*) Periapical radiographs of tooth #30 from patient 5, tooth #21 from patient 6, tooth #5 in patient 15, and tooth C in patient 2. Periapical radiolucency, pulp obliteration, and root bulging are observed. Black arrows indicate extracted/exfoliated teeth. *Primary teeth. ^†^Patient 9 has a triplication within chromosome 13 likely affecting *KLOTHO*.

Five extracted or exfoliated teeth from patients 2, 5, 6, 9, and 15 were analyzed by high‐resolution μCT (Fig. 3*A*,*B*). Compared with matched controls (five teeth from four controls), HFTC teeth exhibited dramatically altered pulpal morphology characterized by obliterated pulp chambers and irregular root canals (Fig. 3*B*,*E*). Altered density distribution was also observed with increased density towards the core of the root (Fig. 3*C*). Overall, HFTC teeth exhibited a sevenfold increase in pulp density (53 ± 58 vs 382 ± 39 mg HA/cm^3^, *p* = 0.0004) and a sevenfold decrease in pulp volume (22 ± 8 vs 3 ± 1 mm^3^, *p* = 0.0088) compared with healthy control teeth (Fig. 3*D*). Cementum and dentin in HFTC teeth were indistinguishable by density, further highlighting root development disturbances; combined cementum and dentin densities were reported. Enamel and dentin/cementum densities were not different between HFTC and controls (Supplementary Information Fig. S3). Available permanent HFTC teeth had limited enamel volume because of deep caries, bruxism, and/or crowns.

Despite pulp obliteration, sensory nerves appeared to be present and functioning. Patients 4, 5, 6, and 15 experienced mild‐to‐severe dental sensitivity, which was likely caused by dental caries in patients 4 and 5 (Fig. 3*E*). Patient 6 did not have active caries and responded normally to electric pulp testing, despite having severe occlusal attrition from bruxism (Fig. 3*E*; Supplementary Information Fig. S2). The cause of generalized dental sensitivity in patient 15 was unknown; her maxillary right first premolar developed a fistula requiring the tooth to be extracted (Fig. 3*E*).

**Fig 4:**
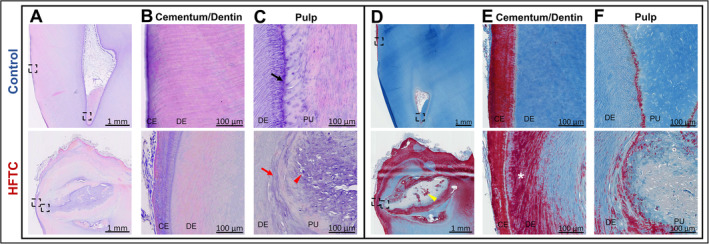
Irregular cellular and structural morphology observed in the dentin, pulp, and cementum of the teeth of patients with hyperphosphatemic familial tumoral calcinosis (HFTC). (*A*) Hematoxylin and eosin (H&E) stain of decalcified control and HFTC (patient 15) teeth. Boxed areas are magnified in (*B*) and (*C*). (*B*) The outer root layer of the control tooth shows normal cementum and dentinal tubules. The outer root layer of the HFTC tooth has similar cementum and dentinal tubules as control. (*C*) The pulp of the control tooth shows organized odontoblasts lining pulp (arrow). Normal pulp contains blood vessels, cells, and nerves. The HFTC tooth is missing the odontoblast layer (arrow). In patients with HFTC, the dental pulp cavity is obliterated and filled by calcified material seen as small rounded elements (psammoma bodies, arrowhead). No soft tissue, capillaries, or odontoblastic cells lining the inner aspect of dentin are detectable. (*D*) Masson's trichrome stain of decalcified control and HFTC (patient 15) teeth. Larger blocks of densely calcified material (yellow arrowhead) are seen in HFTC tooth. Boxed areas are magnified in (*E*) and (*F*). (*E*) Outer root layer of control shows normal cementum in red and collagen in blue. The cementum layer in the HFTC tooth appears relatively normal, but the presence of Sirius red staining in dentinal region (*), which is not seen in the control tooth, suggests the organization of the collagen fibrils in the HFTC tooth is altered. (*F*) Collagen is present in dentin and pulp of control tooth. The relatively lesser degree of aniline blue staining in the HFTC pulp is consistent with less collagen and greater calcification in the pulp. Bulbous expansion of the crown appears to be caused by pulp cavity expansion and calcification. CE, cementum; DE, dentin; PU, pulp.

**Fig 5:**
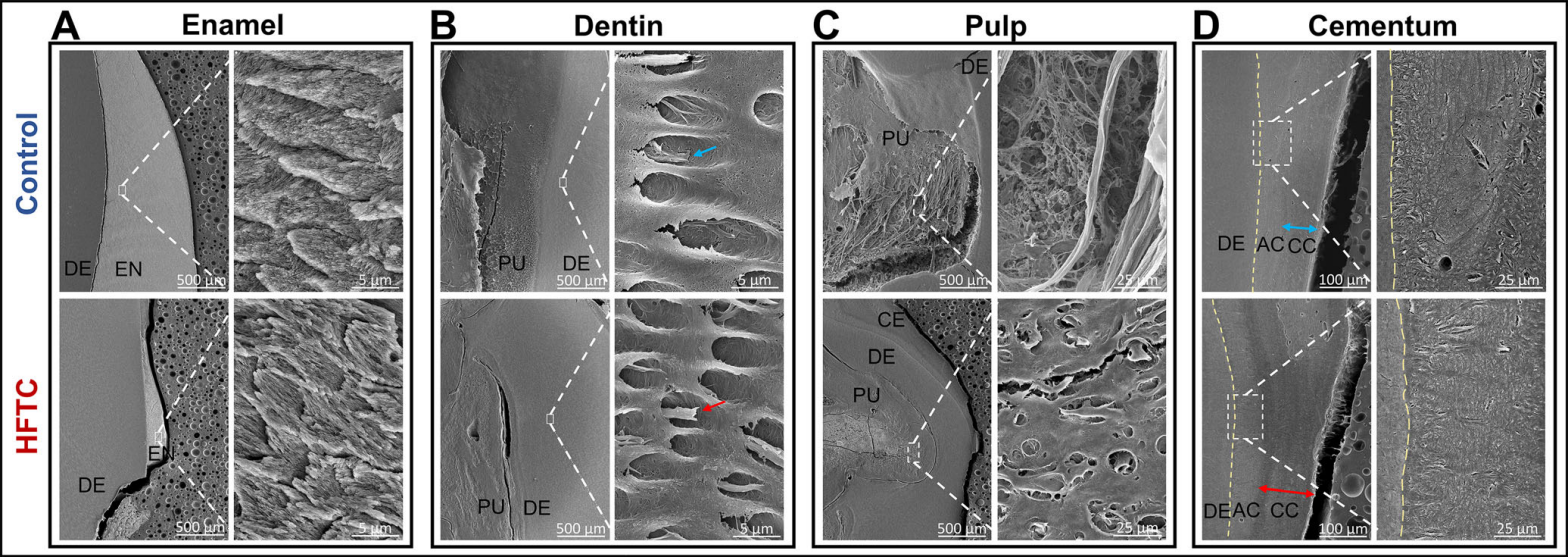
Scanning electron micrographs (SEMs) of control and teeth of a patient with hyperphosphatemic familial tumoral calcinosis (HFTC; patient 15). For each set of panels, low magnification views are on the left and high magnification views are on the right. (*A*) Organized enamel rods are observed in both control and HFTC teeth. (*B*) Normal dentinal tubules and odontoblast processes are observed in control (blue arrow) and HFTC (red arrow) teeth. (*C*) Pulpal cell‐like and fiber‐like structures are observed in control, whereas disorganized dentinal tubules are observed in HFTC pulp. (*D*) Organized acellular and cellular cementum are observed in control teeth. Cellular cementum in HFTC tooth appears to be thicker compared with control. The junction between dentin and acellular cementum is harder to differentiate in the HFTC tooth. Additional SEM images of the pulp are provided in Supplementary Information Figure S4. AC, Acellular cementum; CC, cellular cementum; CE, cementum; DE, dentin; PU, pulp.

Teeth were further examined by histology and scanning electron microscopy (SEM). Histological analyses of a tooth from patient 15 were compared with an adult‐ and tooth‐matched control (Fig. 4*A*–*F*). Analyses revealed relatively normal cementum and dentin layers grossly, histologically (Fig. 4*A*), and by density (Supplementary Information Table S4). However, Masson's trichrome staining identified significant structural differences (Fig. 4*E*), possibly owing to abnormal dentin collagen orientation. The most prominent finding was an obliterated pulp cavity filled with highly dense, calcified material seen as a mix of small rounded elements (psammoma bodies) and larger blocks of calcified material (Fig. 4*C*,*D*). Neither the capillaries nor the normal polarized odontoblastic cell layer lining the inner aspect of dentin were detected in the tooth of the patient with HFTC (Fig. 4*C*, arrows). The widening of the midroot area appeared to be caused by pulp cavity expansion and thickened dentin layer (Fig. 4*A*,*D*). SEM analysis revealed that, compared with controls, the HFTC tooth had normal enamel structure and normal dentin and dentinal tubules with odontoblast processes (Fig. 5*A*–*D*). However, major differences were seen within the pulp and cementum (Fig. 5*C*,*D*). HFTC pulp showed abnormal areas with structures that resembled dentinal tubules (Fig. 5*C*; Supplementary Information Fig. S4), along with a widening of the cellular cementum (Fig. 5*D*).

### HFTC primary teeth also exhibit pulpal alterations

**Fig 6:**
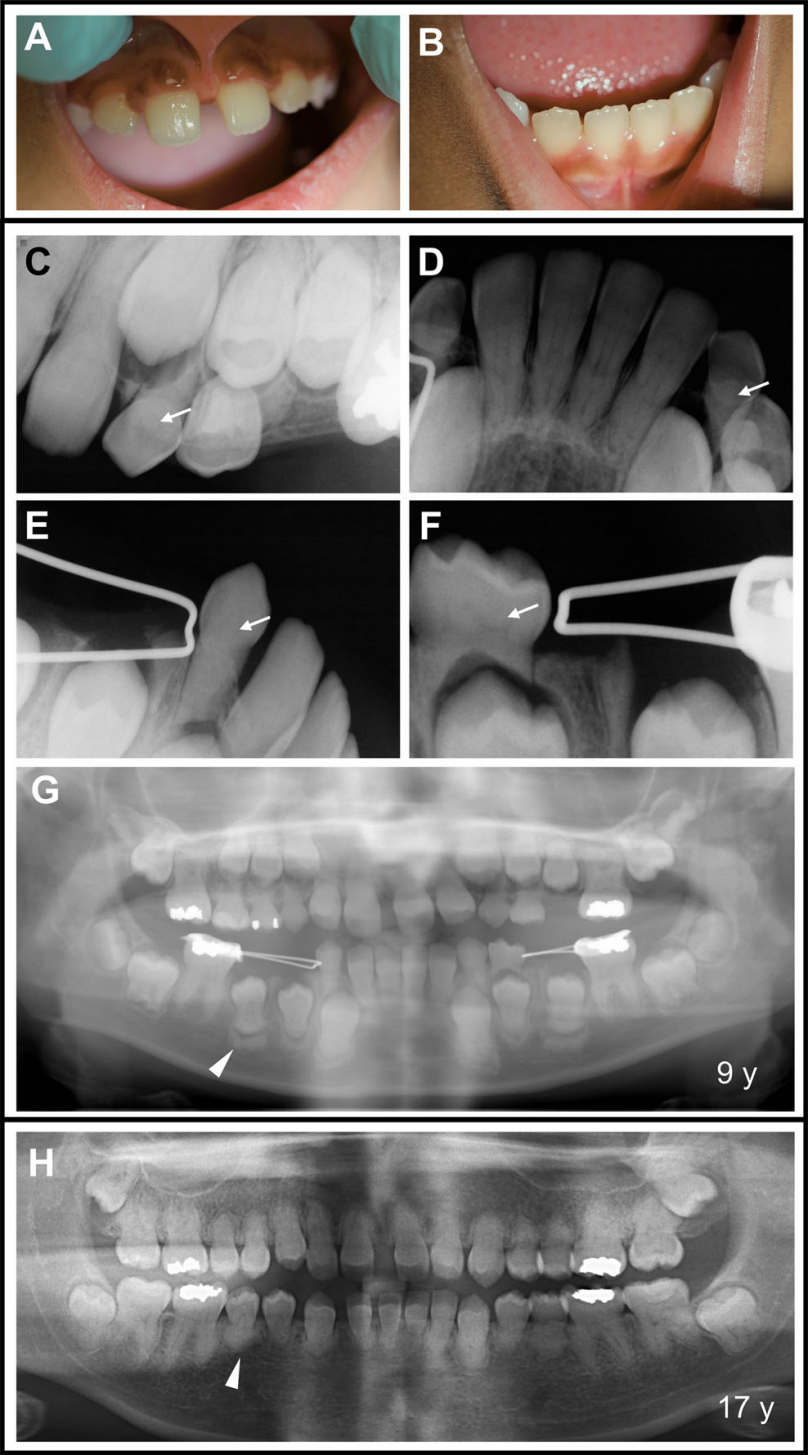
Dental phenotype observed in pediatric patients with hyperphosphatemic familial tumoral calcinosis (HFTC). (*A*–*B*) Clinical oral images of patient 2 at 9 years of age. On visual inspection, the patient did not present any abnormalities. (*C* **–** *G*) However, periapical and panoramic radiographs of patient 2 at 9 years of age reveal pulp calcification (*C* **–** *F*, arrows) in maxillary and mandibular primary teeth. Most of the developing permanent teeth in the mandible show more radiopaque alveolar bone formation around the forming root (*G*, arrowhead) (*H*) Panoramic radiograph of patient 2 at 17 years of age shows severely shortened roots (arrowhead), raising the question if root shortening derives from the altered bone highlighted by the arrowhead in *G*. Calcified pulp is seen in the majority of teeth.

Primary dentition from pediatric patients had findings similar to the mild or moderate phenotypes of HFTC permanent dentition. Patient 2ʼs dental radiographs from 6–17 years of age showed abnormal development (Fig. 6). Although crowns appeared to form normally, signs of pulp calcification during root development were visible at age 6 years. She and her siblings (patients 3 and 14) share the same *GALNT3* mutation. Her younger sister, 15 years old at her most recent visit, showed similar, but milder dental findings. The youngest sibling, at 16 months, was too young for dental radiographs.

Interestingly, patient 10 whose disease was caused by FGF23 autoantibodies also had an abnormal, but a subtly distinct, tooth phenotype compared with other pediatric patients with HFTC (Supplementary Information Fig. S5). Clinically, the teeth appeared normal, but upon radiographical inspection, pulp calcification in primary and developing permanent teeth was observed.

## Discussion

These data represent the most in‐depth analyses to date of dental findings in a large cohort of patients with low or inappropriately normal iFGF23, high cFGF23, and high phosphate. Characteristic findings of thistle‐shaped roots and pulp obliteration appeared at an early age. This could serve as a phenotypic marker of HFTC, offering an opportunity for early diagnosis. Because the findings also occurred in the patient with autoimmune‐mediated disease, it suggests the pathophysiology is not genetic per se, but caused by either a lack of FGF23 or high blood phosphate, suggesting one or both are important for pulp and root development.

### Characteristic dental findings can be used for early HFTC diagnosis

For the first time, we show in‐depth μCT, histological, and SEM analyses of HFTC teeth, showing dramatic replacement of normal pulp with dense, calcified material and altered densities in pulp, dentin, and cementum. Consistent with a recent case report,^(^ ^21^ ^)^ pulp obliteration and internal pulp calcification were observed in our cohort. The presence of normal dentinal tubules with remnants of odontoblastic processes indicates that dentin‐producing cells, odontoblasts, functioned normally during early dental development. Though odontoblasts normally maintain a single layer of highly polarized cell bodies throughout the life of a tooth,^(^ ^22^ ^)^ the odontoblasts in HFTC appear to have lost their polarity and migrated to the center of the pulp. As calcification progressed, some odontoblasts may have become trapped within the disordered and calcified matrix. The calcified mass and hypercementosis in our study parallels findings from a family with HFTC.^(^ ^16^ ^)^ Understanding the dental phenotype aids in identifying pathognomonic signs of HFTC.

Although patients presented with a broad spectrum of clinical symptoms, specific dental findings were consistently present. For example, asymptomatic siblings exhibited characteristic HFTC dental abnormalities before any systemic symptoms developed. Thus, dentists may be the first provider to detect signs of HFTC, which could lead to early interventions and possible reduction of future systemic calcifications.

### Pathophysiology of HFTC dental findings

All patients with *GALNT3* and *FGF23* mutations and FGF23 autoantibodies had what we classify as a characteristic tumoral calcinosis dental phenotype consisting of short, thistle‐shaped roots and pulp calcification. The unifying pathophysiological findings in all were elevated blood phosphate, low or inappropriately normal iFGF23, and elevated cFGF23, or FGF23 inaccessible for binding and signaling due to anti‐FGF23 autoantibodies.^(^ ^12^ ^)^ It has been suggested that high levels of cFGF23 may block iFGF23 signaling,^(23^ ^)^ and for that reason, patients with an iFGF23 in the low‐normal range may have functional loss of iFGF23ʼs capacity to bind and signal. Therefore, it is not possible to discern whether the dental phenotype is attributable to high phosphate or an absence of iFGF23 signaling.

Notably, the patient with a triplication within chromosome 13 likely affecting *KLOTHO* did not have the characteristic dental phenotype. She had a profound neurological deficit that made dental X‐rays impossible; a single exfoliated primary tooth exhibited normal pulp morphology, but modestly increased pulp density, and her blood phosphate was only mildly elevated. Similarly, patient 7, who had normal pulp morphology, did not have a *GALNT3* or *FGF23* mutation, and did not have findings consistent with a *KLOTHO* mutation, had classic calcinosis findings, a markedly elevated cFGF23, and a blood phosphate that was at the upper limit of the normal range.

Mouse models of phosphate and FGF23 disorders have informed the role of inorganic phosphate and FGF23 in development, maintenance, disease, and repair of tooth tissues.^(^ ^24^ ^)^ *Fgf23* ablation in a mouse model resulted in hyperphosphatemia, narrow pulp chambers with ectopic matrix formation, accumulation of osteoid and increased apoptosis in alveolar bone, periodontal ligament space narrowing, and alteration of cementum structure, but did not demonstrate altered root length or shape.^(^ ^25^ ^)^ Conversely, the X‐linked hypophosphatemic (*Hyp*) mouse exhibited increased FGF23 levels, enlarged pulp chambers, widened predentin, interglobular dentin, and irregular dentinal tubule distribution.^(^ ^26^ ^)^ Because phosphate plays a significant role in tooth development and maintenance, the interactions of phosphate and other genes that regulate phosphate^(^ ^27^ ^)^ with other factors, such as calcium, vitamin D, and PTH, are also important considerations. Furthermore, it is likely that additional enzymes, or incompletely understood inorganic molecules, such as pyrophosphate, may play important roles in this complex pathophysiology.

Interestingly, similar, but milder, dental radiographic findings (pulp calcification and short roots) have been reported in pseudohypoparathyroidism,^(^ ^28^ ^)^ another rare inherited hyperphosphatemic disorder. However, the characteristic short, thistle‐shaped roots seen in HFTC have not been reported in pseudohypoparathyroidism, suggesting that elevated phosphate alone is not sufficient to replicate the dental phenotype seen in tumoral calcinosis.

### HFTC tooth defects develop at a young age

In our cohort, and contrary to a previous report,^(^ ^20^ ^)^ pulp obliteration and short roots were also observed in primary teeth. μCT analysis of primary teeth suggests a milder defect in primary versus permanent teeth. Permanent premolar roots were most severely affected, while permanent incisor roots were least affected. This suggests that HFTC affects dental development of permanent tooth roots most severely at ages 9–11 years, when incisors are completing root formation and premolar roots are actively developing. Root development of the premolars start at about 7–8 years of age and continue until about 11–12 years of age. They are the last teeth (other than third molars) to undergo root development. Systemic processes contributing to abnormal root formation appear to peak at the time. HFTC may lead to early exfoliation of primary teeth; all pediatric patients had complete permanent dentition by age 11 years, whereas this process usually occurs up to age 12 years. Once established, the dental phenotype does not appear to significantly change over time, as evidenced by the lack of root severity changes in patient 4ʼs radiographs from 2011–2017 (Supplementary Information Fig. S6), and the fact that older patients did not appear to have a more pronounced dental phenotype compared with younger patients. These data suggest the dental phenotype in HFTC is caused by a primary rather than an acquired defect.

### Clinical significance

With the calcified irregular pulp canals in HFTC, root canal therapy may not be possible. Consequently, dental treatment options for pulpal pathology in patients with HFTC may be limited to extractions. Thus, proper oral hygiene and regular preventive care to reduce the risk of caries and tooth loss is imperative.

Despite altered root morphology, patients with HFTC have successfully undergone oral surgical, periodontal, and orthodontic treatments. Patients 15 and 17 have had dental implants for several years without any associated complications and maintained adequate alveolar bone levels. In addition, several patients have worn orthodontic appliances without reported complications or an increase in external root resorption. This suggests HFTC teeth do not ankylose to the surrounding alveolar bone, allowing adequate teeth movement.

Although this study describes the largest reported HFTC cohort, the sample size remains small because of the rarity of the disease. Because the NIH is a tertiary referral center, referral bias may affect our findings, and the patients may represent the severe end of the spectrum.

This study adds significant new insights into HFTC and tooth development, suggesting a role for blood phosphate and/or FGF23 in root and pulp development. Pulp obliteration is one of the first signs of HFTC and can serve as an early diagnostic marker, allowing for earlier therapeutic intervention. The regulation of pulp formation by phosphate and FGF23 remains an important area warranting further investigation.

## Author contributions

**Alisa Lee:** Conceptualization; data curation; formal analysis; investigation; methodology; project administration; resources; software; validation; visualization; writing‐original draft; writing‐review & editing. **Emily Chu:** Conceptualization; data curation; formal analysis; investigation; methodology; resources; software; supervision; validation; visualization; writing‐review & editing. **Pamela Gardner:** Conceptualization; data curation; formal analysis; investigation; methodology; resources; supervision; validation; visualization; writing‐review & editing. **Olivier Duverger:** Data curation; formal analysis; investigation; methodology; resources; supervision; validation; visualization; writing‐review & editing. **Amanda Saikali:** Formal analysis; investigation; visualization; writing‐review & editing. **Sean Wang:** Formal analysis; investigation; visualization; writing‐review & editing. **Rachel Gafni:** Data curation; formal analysis; investigation; methodology; resources; supervision; validation; visualization; writing‐review & editing. **Iris Hartley:** Data curation; formal analysis; investigation; methodology; supervision; validation; writing‐review & editing. **Kelly Ten Hagen:** Funding acquisition; resources; supervision; writing‐review & editing. **Martha Somerman:** Formal analysis; funding acquisition; resources; supervision; writing‐review & editing.

## Authors' roles

**Alisa Lee:** Study design, data collection, data analysis, data interpretation, writing–original draft. **Emily Chu:** Study design, data collection, data analysis, data interpretation. **Pamela Gardner:** Study design, data analysis, data interpretation. **Olivier Duverger:** Data collection, data analysis, data interpretation. **Amanda Saikali:** Data collection, data analysis. **Sean Wang:** Data collection. **Rachel Gafni:** Data analysis, data interpretation. **Iris Hartley:** Data collection, data analysis, data interpretation. **Kelly Ten Hagen:** Data interpretation. **Martha Somerman:** Data interpretation. **Michael Collins:** Study design, data interpretation, writing–original draft. The manuscript was reviewed, edited, and approved by all authors.

## Conflict of Interest

The authors declare no potential conflicts of interest with respect to the authorship and/or publication of this article.

### Peer review

The peer review history for this article is available at https://publons.com/publon/10.1002/jbm4.10470.

## Supporting information


**Appendix S1**: Supporting information
