# Supplementary material for: A Cross‐Sectional Cohort Study of the Effects of FGF23 Deficiency and Hyperphosphatemia on Dental Structures in Hyperphosphatemic Familial Tumoral Calcinosis
Source: JBMR Plus. 2021 Mar 22;5(5):e10470. doi: 10.1002/jbm4.10470 (PMC8101615; doi:10.1002/jbm4.10470)
Supplement: Supplementary file 1 — Appendix S1: Supporting information [file JBM4-5-e10470-s001.docx]

**SUPPLEMENTAL MATERIAL**

**
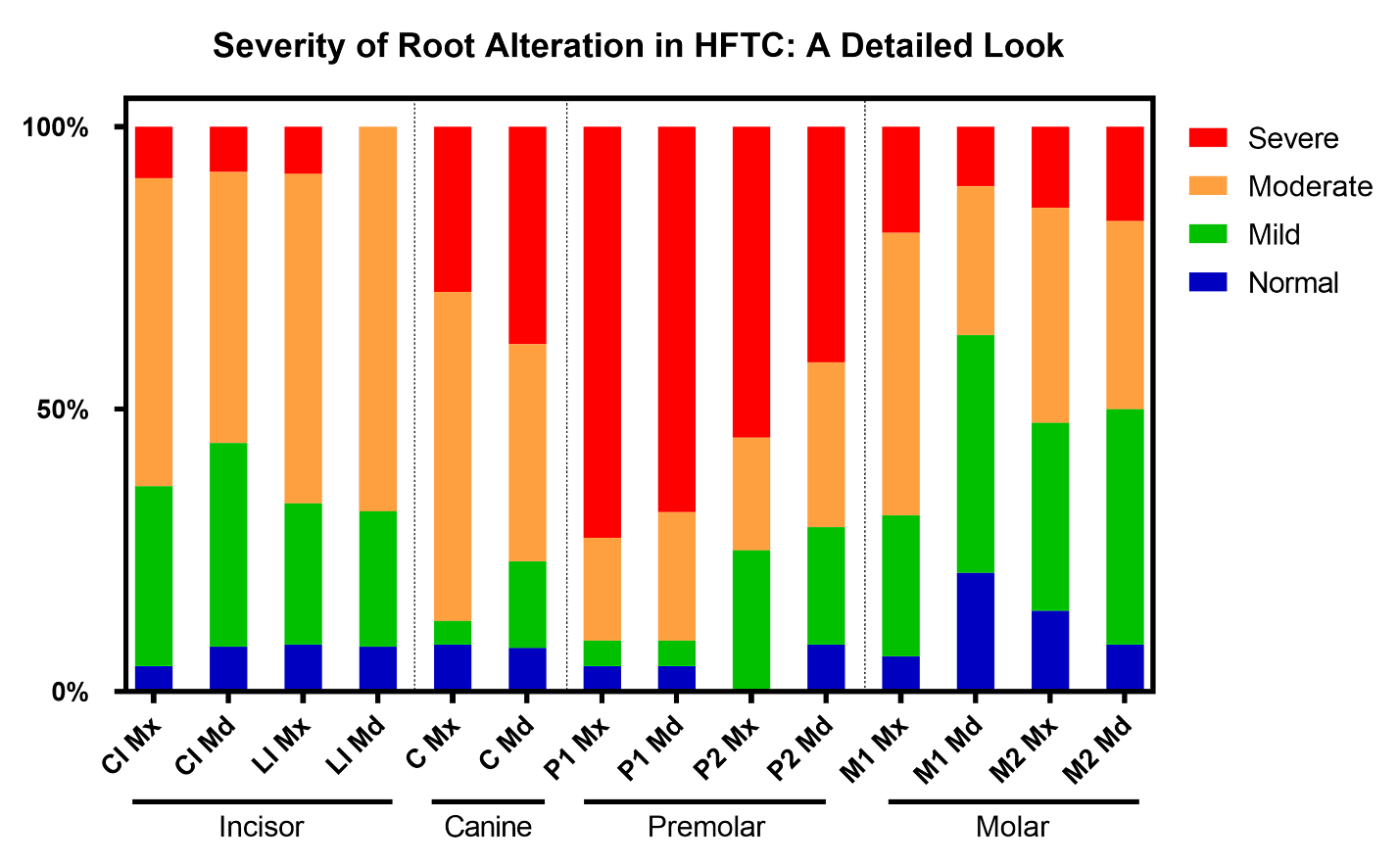
**

**Supplemental Figure S1.** A detailed determination of the severity of root morphology based on tooth type. Premolars are most severely affected, especially maxillary and mandibular first premolars. Mx = maxillary, Md = mandibular, CI = central incisor, LI = lateral incisor, C = canine, P1 = first premolar, P2 = second premolar, M1 = first molar, M2 = second molar.


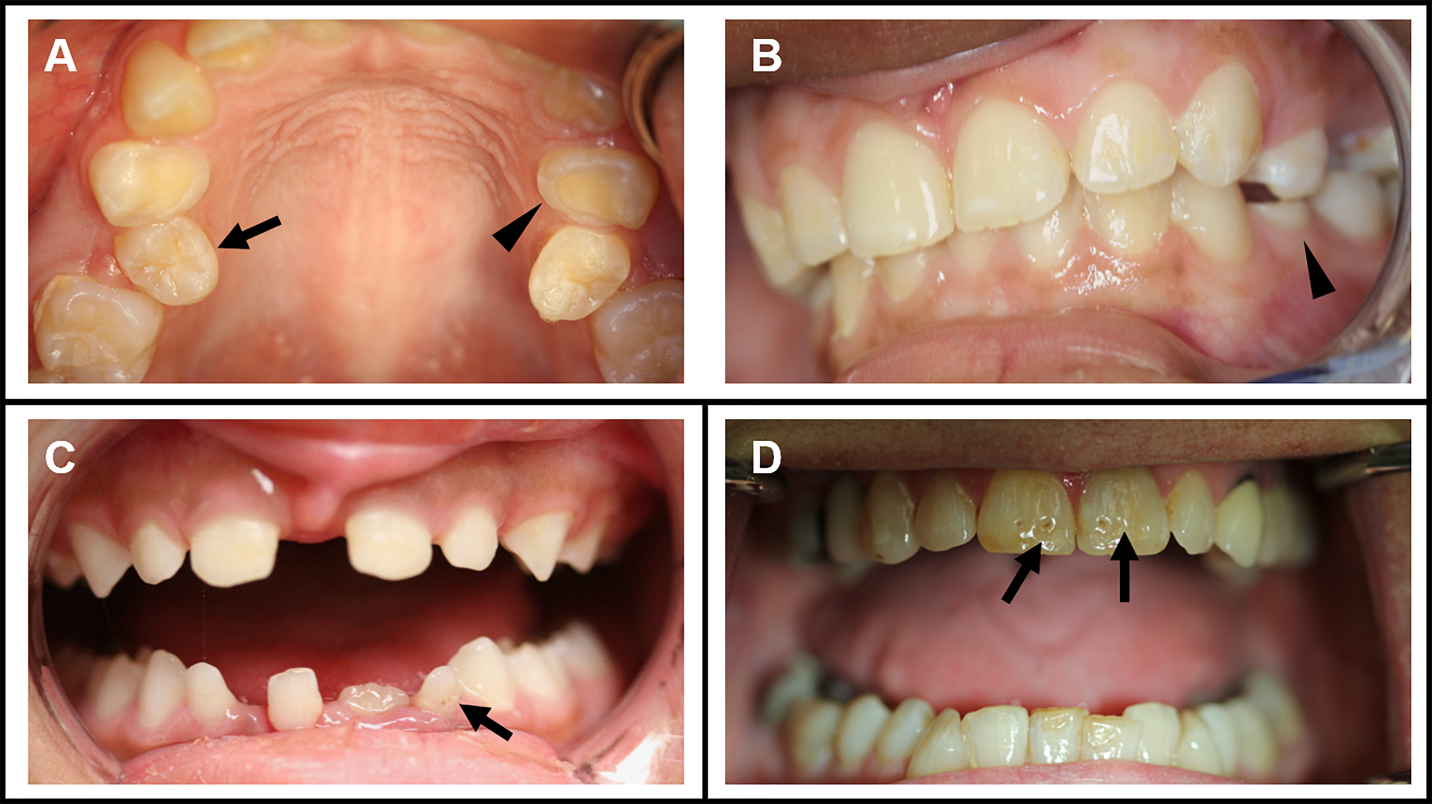


**Supplemental Figure S2.** Intraoral examination of some HFTC patients reveal enamel defects. (**A–B**) Patient 2 at age 12 presents white chalky enamel (arrow) and severe occlusal attrition from bruxism (arrowheads). (**C**) Patient 6 at age 6 presents white chalky enamel (arrow), possibly due to mild fluorosis. (**D**) Patient 7 at age 56 presents moderate localized enamel hypoplasia and pitting of facial surface (arrows). Enamel show yellow and gray discoloration.


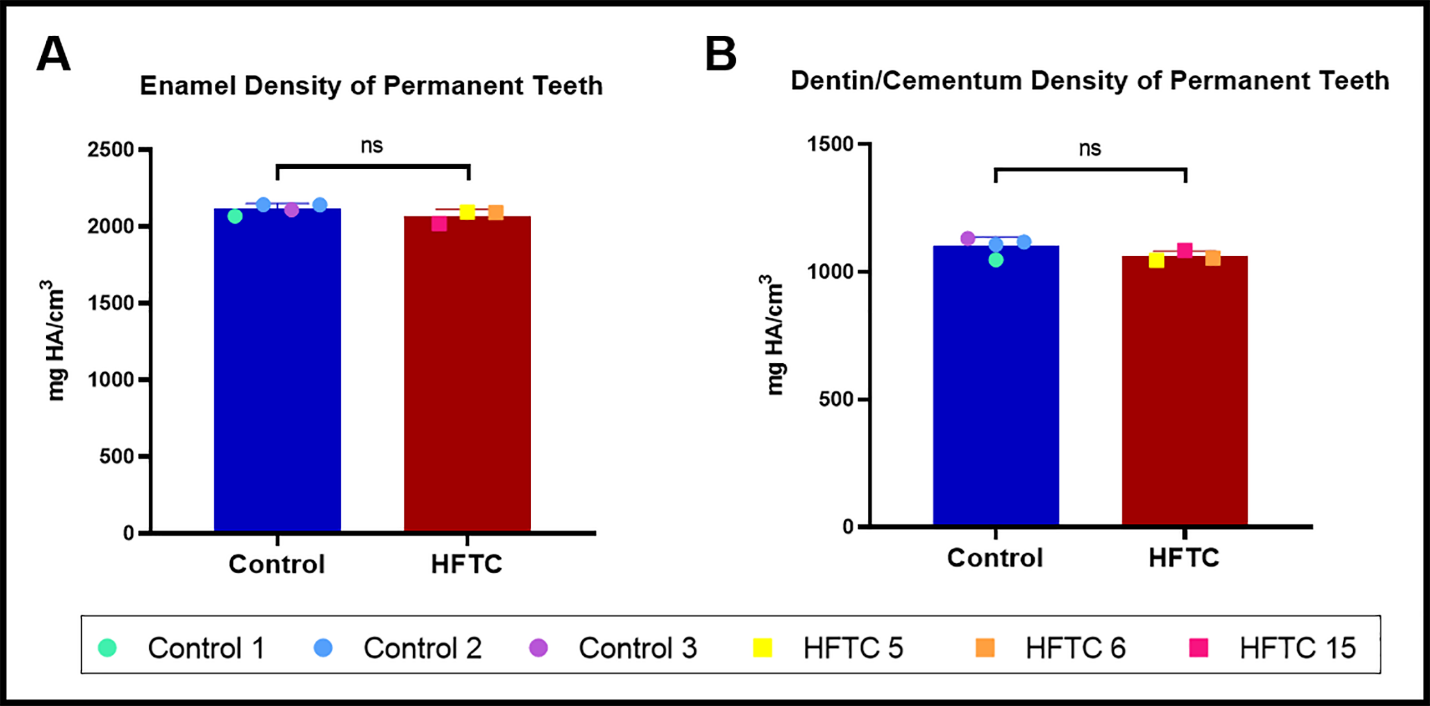


**Supplemental Figure S3.** Permanent HFTC teeth have normal enamel (**A**) and dentin/cementum (**B**) densities when compared to age- and tooth-number-matched healthy control teeth.


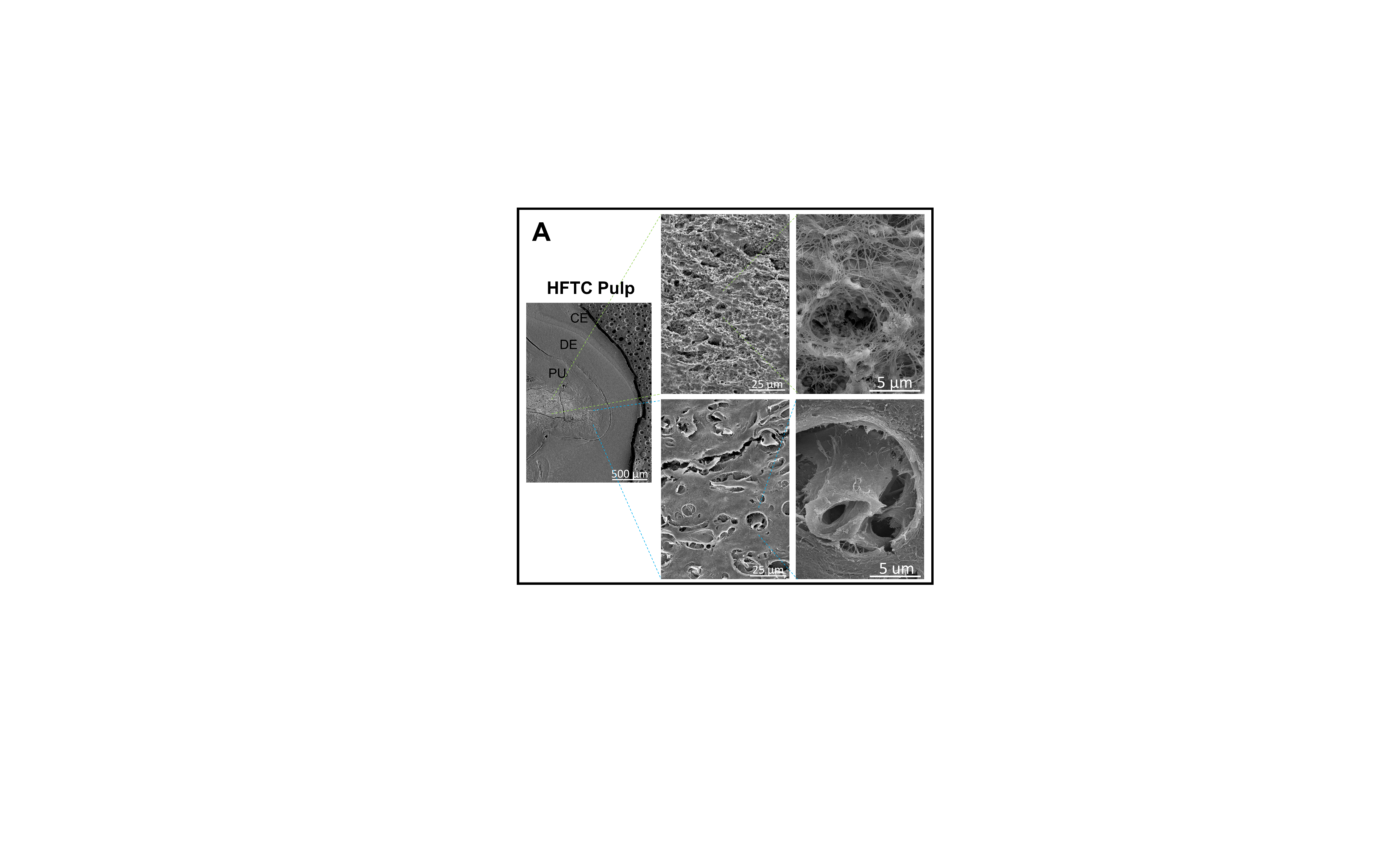


**Supplemental Figure S4.** Scanning electron micrographs (SEMs) show a heterogeneous mixture in patient tooth pulp. (**A**) Low magnification of pulp chamber in the tooth from patient 15. High magnification of the center area in pulp chamber shows fiber like structures and cells. Periphery of pulp chamber shows structures that resemble abnormal dentinal tubules in control (Fig. 3G). DE = dentin, PU = pulp, CE = cementum.


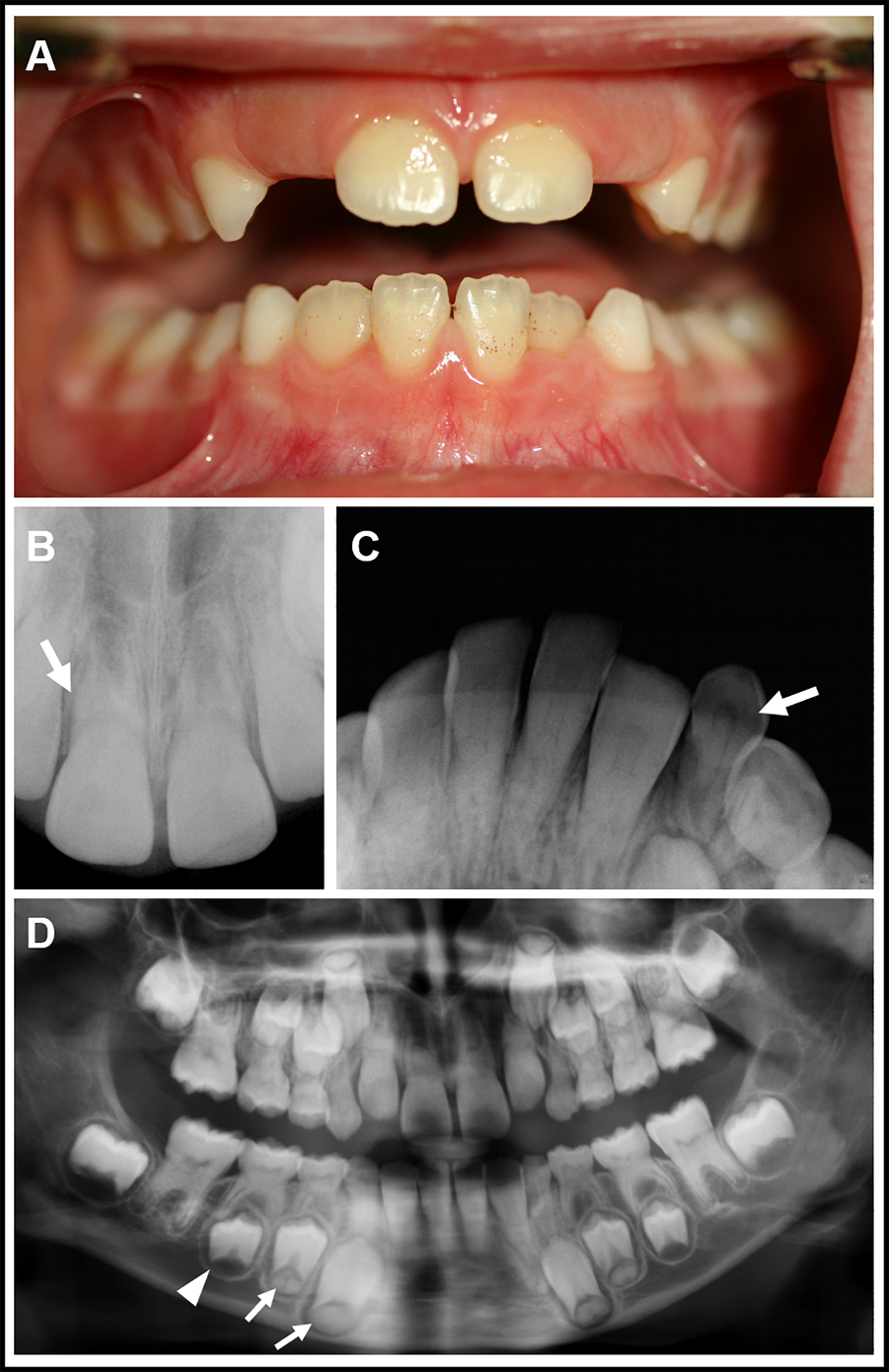


**Supplemental Figure S5.** Dental phenotype observed in an autoimmune pediatric HFTC patient. (**A**) Clinical oral image of patient 10 at age 7. The patient was found to have neutralizing autoantibodies to fibroblast growth factor 23 (FGF23) (Roberts et al. 2018). (**B**) A periapical radiograph of maxillary central incisors shows root bulging and pulp calcification (arrow). (**C**) A periapical radiograph of mandibular incisors and canine. Primary canine teeth show pulp calcification (arrow). (**D**) A panoramic radiograph of patient 10 at age 7. An abnormal presentation of developing permanent teeth is distinct from what was observed in patient 2 (Fig. 4). Mandibular second premolars appear to have normal development (arrowhead), while first premolars and canines show abnormal calcification in the developing pulp (arrows).


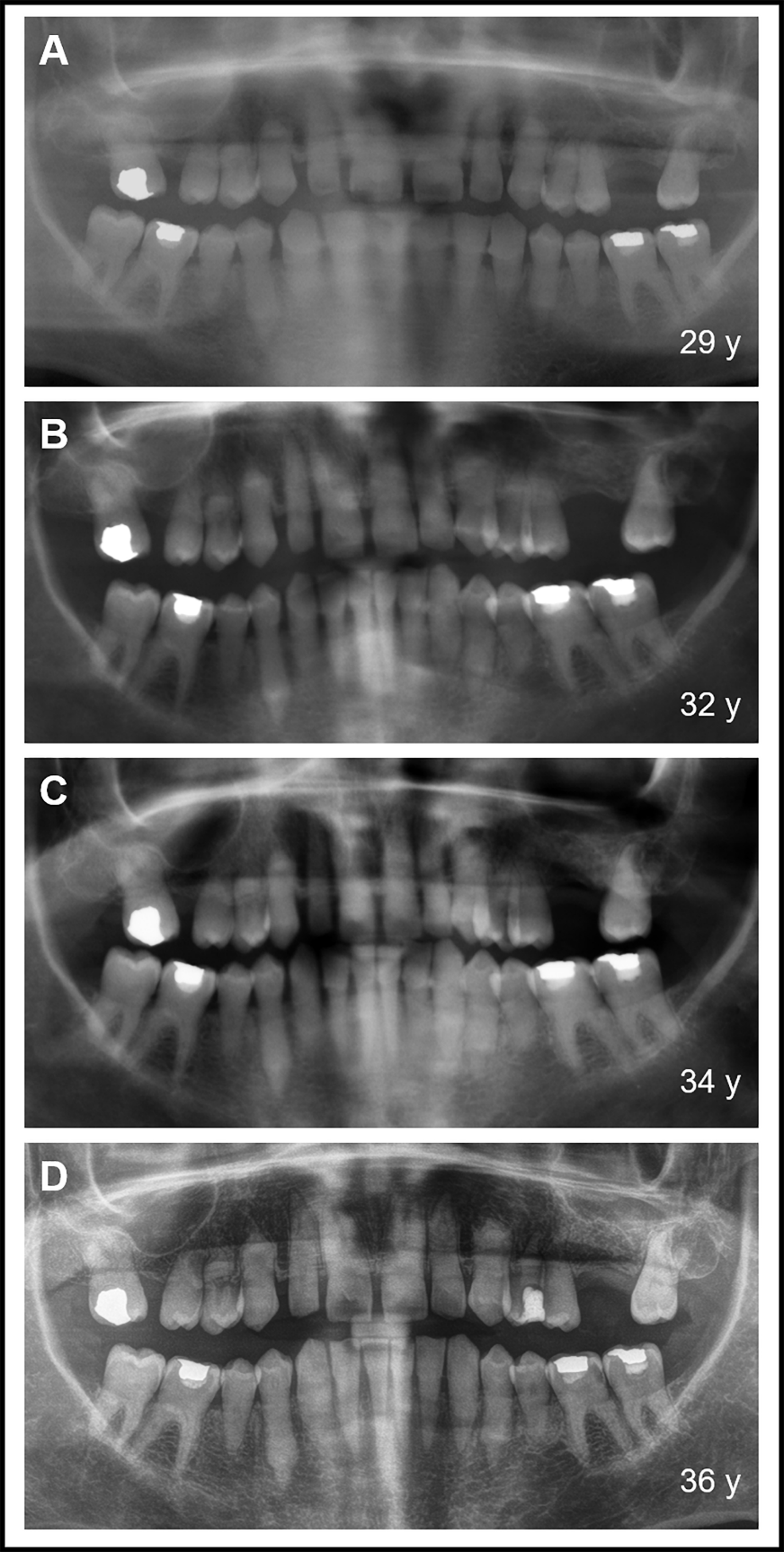


**Supplemental Figure S6.** Panoramic radiographs of patient 4 from 2011 to 2017. No significant changes in tooth severity is noted between radiographs from (**A**) 29 years old, (**B**) 32 years old, (**C**) 34 years old, and (**D**) 36 years old, suggesting relative stability of the findings at this age.

**Supplemental Table S1.** Genes Involved in Hyperphosphatemic Familial Tumoral Calcinosis (HFTC).

| **Gene** | **NCBI Reference Sequence** | **OMIM** | **Phenotype MIM Number** | **Location** |
| --- | --- | --- | --- | --- |
| *GALNT3* | NM_004482.4 | 601756 | 211900 | 2q24.3 |
| *FGF23* | NM_020638.3 | 605380 | 617993 | 12p13.32 |
| *KLOTHO* | NM_004795.4 | 604824 | 617994 | 13q13.1 |

GALNT3, UDP-GalNAc:polypeptide N-acetylgalactosaminyltransferase 3; FGF23, Fibroblast Growth Factor 23; NCBI, National Center for Biotechnology Information; OMIM, Online Mendelian Inheritance in Man.

**Supplemental Table S2.** Genetic Findings in HFTC Cohort

| **Patient** | **Gene** | **DNA change** | **Protein change** |
| --- | --- | --- | --- |
| 1 | *GALNT3* | c.1312C>T, c.1774C>T | p.(Arg438Cys), p.(Gln592Ter) |
| 2^a^ | *GALNT3* | c.516-2A>T, c.260_266del | p.?, p.(Arg87ThrfsTer19) |
| 3^a^ | *GALNT3* | c.516-2A>T, c.260_266del | p.?, p.(Arg87ThrfsTer19) |
| 4^b^ | *GALNT3* | c.1584dup | p.(Pro529ThrfsTer17) |
| 5^b^ | *GALNT3* | c.1584dup | p.(Pro529ThrfsTer17) |
| 6 | *GALNT3* | c.516-2A>T, c.1524+5G>A | p.?, p.? |
| 7 | Unknown | n/a | n/a |
| 8 | *GALNT3* | c.746_749del, c.892del | p.(Val249AspfsTer8), p.(Tyr298ThrfsTer5) |
| 9 | *KLOTHO* | arr 13q13.1q14.3(32,887,503-53,157,340)x4 dn^c^ | Triplication |
| 10 | Autoimmune | n/a | n/a |
| 11 | *GALNT3* | c.766G>C^c^ | p.(Gly256Arg) |
| 12 | *FGF23* | c.211A>G, c.107G>A^c^ | p.(Ser71Gly), p.(Trp36Ter) |
| 13^b^ | *GALNT3* | c.1584dup | p.(Pro529ThrfsTer17) |
| 14^a^ | *GALNT3* | c.516-2A>T, c.260_266del | p.?, p.(Arg87ThrfsTer19) |
| 15 | Unknown | n/a | n/a |
| 16 | *GALNT3* | c.985G>A^c^, c.1677_1680dup^c^ | p.(Gly329Arg), p.(Cys561IlefsTer71) |
| 17 | *GALNT3* | c.746_749del, c.926T>G^c^ | p.(Val249AspfsTer8), p.(Ile309Arg) |

GALNT3, UDP-GalNAc:polypeptide N-acetylgalactosaminyltransferase 3; FGF23, Fibroblast Growth Factor 23; n/a, not available.

^a^ Patients 2, 3, and 14 are siblings.

^b^ Patients 4, 5, and 13 are siblings.

^c^ Novel mutation.

**Supplemental Table S3.** Dental Findings in HFTC Cohort.

| **Patient** | **Root alteration severity^a^** | **Short root length** | **Pulp calcification** |
| --- | --- | --- | --- |
| 1 | 3.1 | Yes | Yes |
| 2^b^ | 3.4 | Yes | Yes |
| 3^b^ | 2.5 | Yes | Yes |
| 4^c^ | 2.6 | Yes | Yes |
| 5^c^ | 3.2 | Yes | Yes |
| 6 | 3.7 | Yes | Yes |
| 7 | 1.0 | WNL | WNL |
| 8 | n/a | n/a | n/a |
| 9 | n/a | n/a | n/a^e^ |
| 10 | n/a | n/a | Yes |
| 11 | 3.4 | Yes | Yes |
| 12 | 3.3 | Yes | Yes |
| 13^c^ | 3.0 | Yes | Yes |
| 14^b^ | n/a | n/a | n/a |
| 15 | 2.6 | Yes | Yes |
| 16 | 2.7 | Yes | Yes |
| 17 | 3.1 | Yes | Yes |
| **Range^d^** | 1.0 – 3.7 | **Prevalence^d^** | |
| **Mean^d^** | 2.9 | 12/13 | 12/13 |
| **Median^d^** | 3.1 |  |  |

WNL, within normal limits; n/a, not available.

**^a^** Root alteration score is an average of assigned scores based on Figure 1A (1 = Normal, 2 = Mild, 3 = Moderate, 4 = Severe).

^b^ Patients 2, 3, and 14 are siblings.

^c^ Patients 4, 5, and 13 are siblings.

^d^ Calculated excluding patients 9 and 10.

^e^ Dental radiographs were not available. An exfoliated primary tooth had normal pulp morphology, but increased pulp density (200.8 mg HA/cm^3^) by micro-CT analysis.

**Supplemental Table S4.** Density Measurements of Segmented Teeth Layers.

|  | **Control** | | **HFTC (Patient 15)** | |
| --- | --- | --- | --- | --- |
|  | **Volume (mm^3^)** | **Density**  **(mg HA/cm^3^)** | **Volume (mm^3^)** | **Density**  **(mg HA/cm^3^)** |
| **Enamel** | 118 | 2110 | 4 | 2020 |
| **Pulp** | 13 | 142 | 1 | 422 |
| **Dentin** | 360 | 1150 | 80 | 1191 |
| **Cementum** | 53 | 1011 | 122 | 1015 |
| **Combined**  **Dentin & Cementum^a^** | 413 | 1132 | 202 | 1085 |
| **Composite** | n/a | n/a | 17 | 2598 |

n/a, not available.

**^a^** Dentin and cementum in HFTC teeth were indistinguishable by density. Combined dentin and densities were reported.
